# A Case Report of Surgical Resection of Primary Retroperitoneal Squamous Cell Carcinoma and Postoperative Local Recurrence

**DOI:** 10.7759/cureus.107929

**Published:** 2026-04-28

**Authors:** Satoshi Ishida, Akinori Sekioka, Shuichi Ota, Chisa Shibata, Shusuke Yasuoka

**Affiliations:** 1 Gastroenterological Surgery, Osaka Saiseikai Noe Hospital, Osaka, JPN

**Keywords:** primary, recurrence, resection of recurrence, retroperitoneal, squamous cell carcinoma

## Abstract

Primary retroperitoneal squamous cell carcinoma (SCC) is an exceptionally rare malignancy. Here, we present the case of a 55-year-old female with a retroperitoneal tumor who underwent complete surgical resection. Histopathological examination confirmed SCC with no evidence of an alternative primary site, leading to a diagnosis of primary retroperitoneal SCC. Local recurrence in the aortocaval area was detected seven months postoperatively. The second surgical resection achieved complete removal, and the patient has remained disease-free for over four years without further chemotherapy or radiotherapy. This is the first case report of complete surgical resection of a locally recurrent retroperitoneal SCC without additional treatment, leading to a better prognosis.

## Introduction

Retroperitoneal tumors are rare neoplasms accounting for only 0.2% of all tumors [1]. While most cases arise from soft tissue, epithelial tumors of primary retroperitoneal origin have rarely been reported [2]. When squamous cell carcinoma (SCC) occurs in the retroperitoneum, key considerations include determining whether it is primary or metastatic, and if primary, identifying the type of cells that serve as the site of origin.

Although the etiology of primary retroperitoneal SCC remains unclear, hypotheses include squamous metaplasia of embryonic rests, human papillomavirus (HPV) infection, and malignant transformation of endometriosis [2-4]. Complete surgical resection is currently considered the cornerstone of treatment; however, standardized management strategies are lacking, and there is a significant gap in evidence regarding the treatment of local recurrence [3,5,6].

In this report, we present a patient with primary retroperitoneal SCC who underwent surgical resection of the primary tumor and local recurrence.

## Case presentation

**Figure 1:**
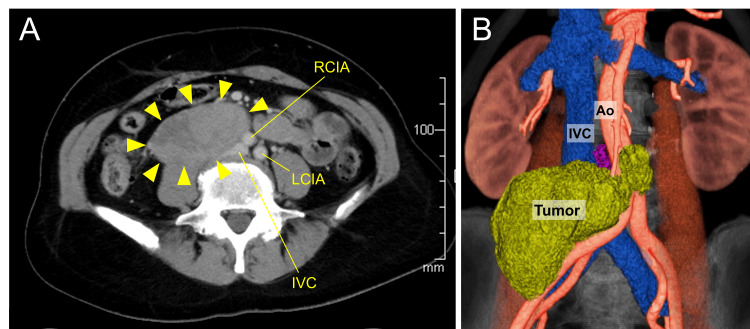
Contract-enhanced computed tomography (CT) showing retroperitoneal tumor (A) Axial view of CT showed a 5 × 4 × 7.5 cm iso-density mass in the right retroperitoneum (arrowheads). (B) On 3D reconstruction images of CT, the Ao and IVC are compressed by the tumor. Ao: aorta, IVC: inferior vena cava, LCIA: left common iliac artery, RCIA: right common iliac artery

A 55-year-old female patient, with a history of endometriosis in her 30s, hypertension, and cervical lymph node tuberculosis, initially presented with mild abdominal pain and fever. Although her symptoms improved naturally, computed tomography (CT) revealed a large mass (5 × 4 × 7.5 cm) located in the retroperitoneal cavity of her right lower abdomen, compressing the inferior vena cava (IVC) and abdominal aorta (Figures 1A-1B). Laboratory findings showed an interleukin-2 receptor level of 835 U/mL (reference range, 122-496), and the carbohydrate antigen 125 level was 103.7 U/mL (reference range, 0-35). Other tumor markers, including carcinoembryonic antigen, carbohydrate antigen 19-9, and duodenal pancreatic cancer antigen 2, were within normal limits. Serum SCC antigen levels measured postoperatively were within the normal range. In addition, urinary metanephrine and normetanephrine levels were within normal limits. Preoperative and postoperative laboratory findings are summarized in Table 1.

**Table 1:** Laboratory data Reference ranges for urinary metanephrine and normetanephrine are not established for spot urine samples. ALT: alanine aminotransferase, AST: serum aspartate aminotransferase, BUN: blood urea nitrogen, CA125: carbohydrate antigen 125, CA19-9: carbohydrate antigen 19-9, CEA: carcinoembryonic antigen, Cl: chlorine, CRP: C-reactive protein, DUPAN2: duodenal pancreatic cancer antigen 2, HCT: hematocrit, HGB: hemoglobin, HPV: human papilloma virus, IL-2 receptor: interleukin-2 receptor, K: potassium, Na: sodium, PCT: procalcitonin, RBC: red blood cell, SCC: squamous cell carcinoma, WBC: white blood cell

Test (Unit)	Result	Reference Range
Preoperative data		
WBC (×10^3^/μL)	8.5	3.5-8.0
RBC (×10^6^/μL)	4.69	3.8-4.8
HGB (g/dL)	14.1	11.3-14.9
HCT (%)	42.1	36.0-47.0
Platelets (×10^3^/μL)	395	120-400
CRP (mg/dL)	0.28	<0.3
Total protein (g/dL)	7.9	6.5-8.2
Serum albumin (g/dL)	4.1	3.7-5.5
AST (IU/L)	21	10-40
ALT (IU/L)	13	5-45
BUN (mg/dL)	12.6	8.0-20.0
Serum creatinine (mg/dL)	0.68	0.5-0.8
Na (mEq/L)	138	136-148
K (mEq/L)	4.2	3.6-5.0
Cl (mEq/L)	102	97-108
IL-2 receptor (U/mL)	835	122-496
CEA (ng/mL)	1.8	<5.0
CA19-9 (U/mL)	26.1	<37.0
CA125 (U/mL)	103.7	<35
DUPAN2 (U/mL)	25	<150
Urinary metanephrine (mg/L)	0.03	-
Urinary normetanephrine (mg/L)	0.08	-
Postoperative data		
SCC (ng/mL)	0.5	<1.5
HPV-DNA	Negative	Negative

**Figure 2:**
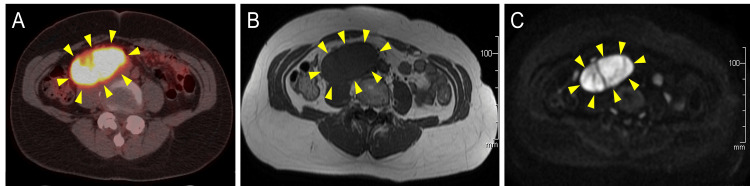
Positron emission tomography (PET) and magnetic resonance imaging (MRI) showing a retroperitoneal tumor (A) PET showed the mass with a maximum standardized uptake value of 21 (arrowheads). (B) MRI showed an isointense signal on T2-weighted images (arrowheads). (C) MRI showed high signal on the diffusion-weighted image (arrowheads).

Esophagogastroduodenoscopy and total colonoscopy revealed no abnormalities. Positron emission tomography (PET) revealed a mass with a maximum standardized uptake value (SUV) of 21 (Figure 2A). Magnetic resonance imaging revealed an isointense signal on T2-weighted images and a high signal on diffusion-weighted images (Figures 2B-2C). Based on these findings, the differential diagnoses were neurogenic tumors, paraganglioma, malignant lymphoma, and Castleman disease. However, because a definitive diagnosis could not be established through this comprehensive preoperative workup alone, a clinical decision was made to proceed with laparotomy. The primary objective was to obtain a histological diagnosis via excisional biopsy and, if feasible, perform a complete therapeutic resection.

**Figure 3:**
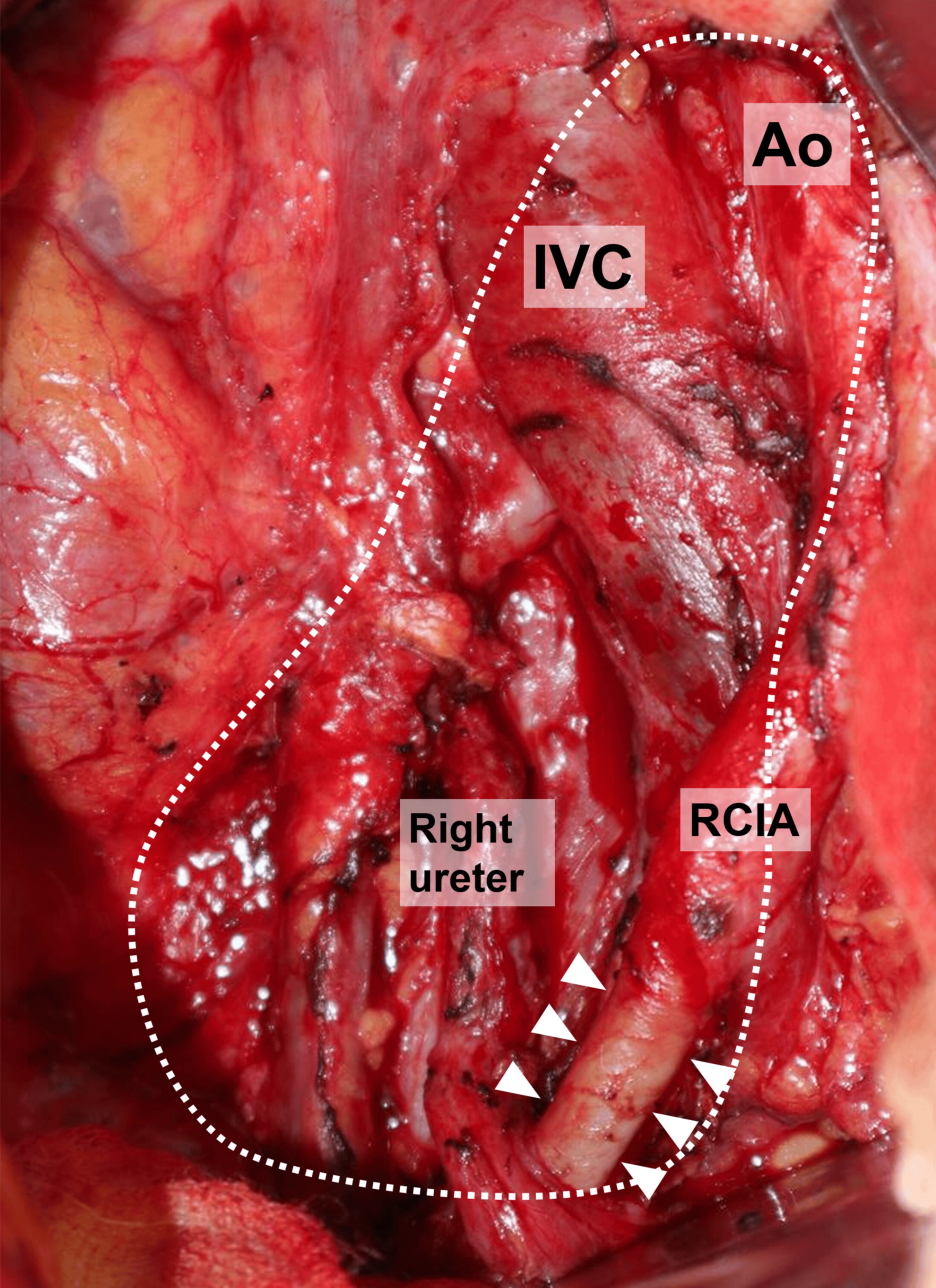
Intraoperative view showing the site after the resection of the primary tumor (dotted line) A portion of the vascular sheath of the right common iliac artery was resected en bloc because of the severe adhesion (arrowheads). Ao: aorta, IVC: inferior vena cava, RCIA: right common iliac artery

**Figure 4:**
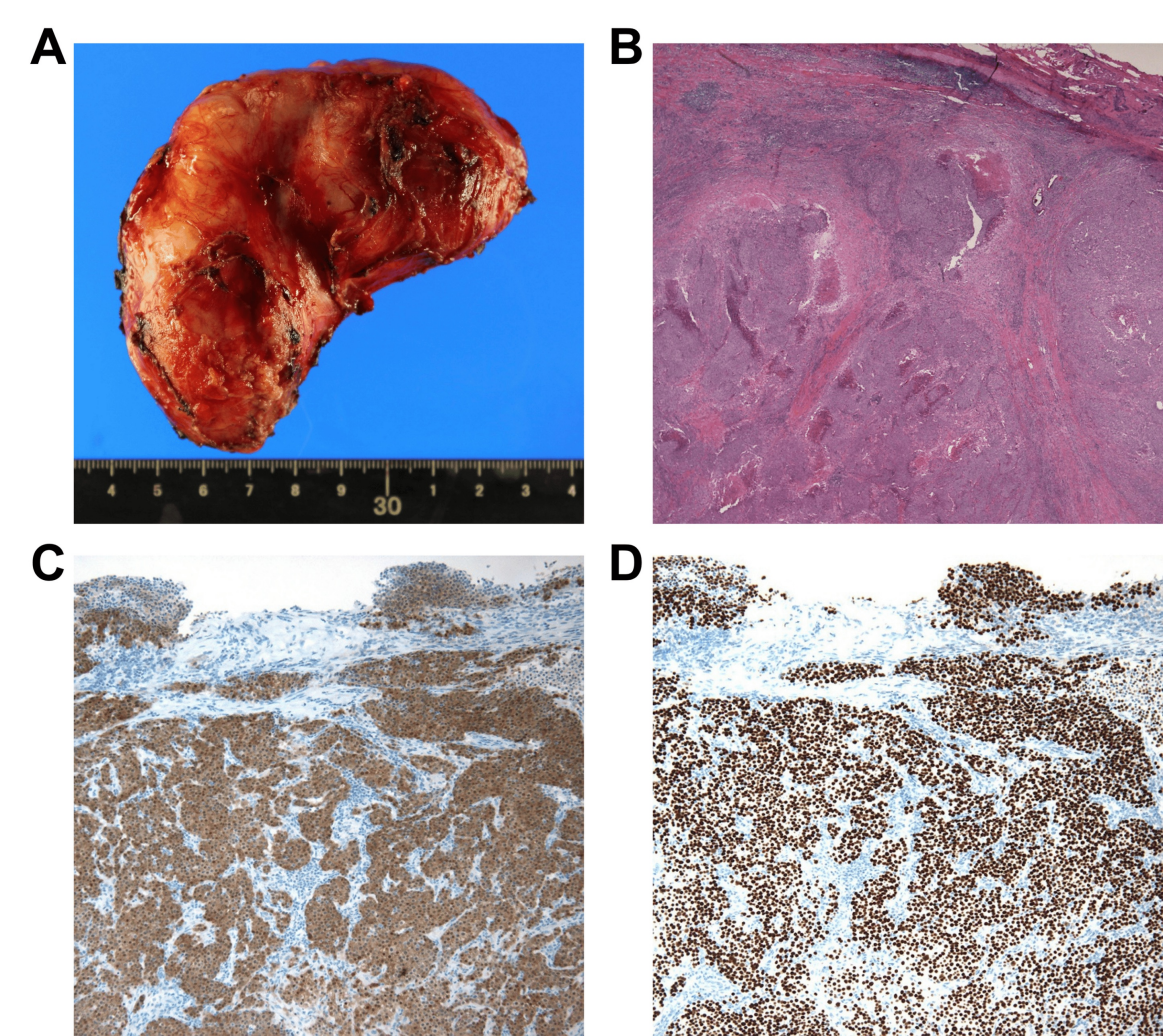
Histopathological findings of the primary retroperitoneal squamous cell carcinoma (A) The tumor was an 8 × 7.5 × 4 cm firm and elastic mass, covered with a fibrous capsule. (B) Histopathological examination revealed an epithelial malignant tumor. (C, D) Immunohistochemical analysis showed positivity for p16 and p40, respectively.

The primary surgery revealed that the mass was located in the retroperitoneal space and was pushing the right ureter ventrally. It adhered to the right ureter, IVC, and right common iliac artery but did not invade. Therefore, the mass was completely removed without damaging other organs (Figure 3). The operative time was 262 minutes, and the intraoperative blood loss was 58 mL. The postoperative course was uneventful, and the patient was discharged on postoperative day 6. Histopathological examination suggested epithelial malignancy with SCC. Immunostaining was positive for p40 and p16 but negative for nuclear protein in the testis. Ki-67 expression was 58% (Figures 4A-4D). Diffuse positivity for p16 suggested that the primary organs were the middle pharynx or cervix.

Given the pathological findings, further evaluations were conducted by the otolaryngology and gynecology departments to identify the primary tumor; however, no abnormalities were detected in the pharynx, uterus, or adnexa. Serum HPV DNA test results were negative. Therefore, we diagnosed SCC of primary retroperitoneal origin. As there were no signs of residual carcinoma based on the radiological and intraoperative findings, the patient was followed up without any additional treatment.

**Figure 5:**
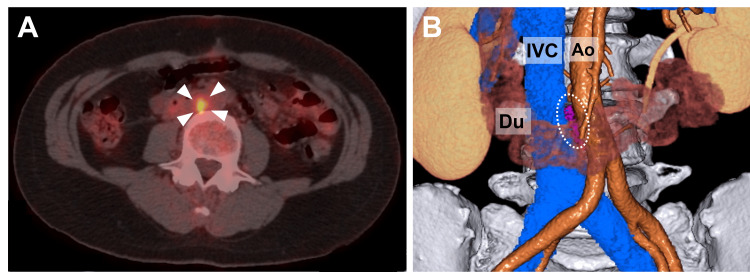
Positron emission tomography (PET) and 3D-computed tomography (CT) showing the local recurrence (A) PET revealed a small mass (1.3 × 0.8 cm) with a maximum standardized uptake value of 6.7 (arrowheads). (B) On 3D-CT reconstructed imaging, the tumor was located in the para-aortic region near the previous surgical site (dotted line). Ao: aorta; Du: duodenum; IVC: inferior vena cava

Seven months after surgery, follow-up CT showed a small mass (1.3 × 0.8 cm) in the aortocaval area near the site where the primary tumor was located. PET showed a mass with a maximum SUV of 6.7 and no evidence of distant metastasis (Figures 5A-5B). Local recurrence was determined, and surgical treatment was performed. The tumor was identified on the lumbar vertebrae between the abdominal aorta and IVC after mobilizing the duodenum. It adhered to the aorta and IVC but was completely resected without damage to other organs. The operative time was 189 minutes, and the intraoperative blood loss was 150 mL. The postoperative course was uneventful, and the patient was discharged on postoperative day 6.

Histopathological findings showed that the tumor cell type was SCC, which is consistent with local recurrence of primary retroperitoneal SCC. Because there were no signs of distant metastasis, additional treatment was not performed after careful discussion with the patient.

The patient has remained in good condition, with no recurrence for over four years after the second operation.

## Discussion

The retroperitoneum originates from the mesoderm, and retroperitoneal tumors are predominantly non-epithelial, with epithelial tumors being relatively rare. The origin and etiology of retroperitoneal SCC remain unknown. Additionally, some reports suggest the involvement of HPV in retroperitoneal SCC [5,6], whereas others indicate the possibility of developing SCC based on endometriosis [4].

In the pathological examination of this case, only pure SCC components were observed, with no evidence of endometriosis. Furthermore, no abnormalities were detected in the peritoneal cavity, uterus, or adnexa, effectively ruling out the possibility of tumor development from endometriosis or metastasis from gynecological cancer. Comprehensive systemic evaluation failed to identify the primary lesion, leading to the diagnosis of primary retroperitoneal SCC. Immunohistochemical analysis revealed positive staining for p16; however, HPV was less likely to be involved in this tumor because of a negative HPV DNA test.

Previous studies have revealed several cases of SCC that were considered to be retroperitoneal in origin, as summarized in Table 2 [2-11]. The most common age was approximately 50 years, and most patients were female. Half of the patients were diagnosed using CT or ultrasound-guided biopsy, and the other half underwent exploratory laparotomy before confirming the diagnosis. Curative surgery was performed in nine patients, with postoperative therapy (chemotherapy, radiation, or concomitant chemoradiotherapy) administered in most of them. Among the patients who underwent curative resection, three experienced recurrence, two local recurrences, and one supraclavicular lymph node recurrence. One patient with local recurrence was treated with chemotherapy (taxane and platinum), whereas another patient did not undergo further treatment and died six months after surgery.

**Table 2:** Summary of previously reported cases of primary retroperitoneal squamous cell carcinoma AWD: alive with disease; CRT: chemoradiotherapy; CT: chemotherapy; DOD: dead of disease; NED: no evidence of disease

Case	Author (Year)	Age/Sex	Initial Treatment	Recurrence	Treatment for Recurrence	Outcome (Follow-Up)
1	Khalil et al. [10] (2005)	57F	Surgery + CT (PF)	Local	None	DOD (6 months)
2	Clements et al. [9] (2010)	34F	CRT (cisplatin)	Cervix	Multiple CT/targeted	AWD (23 months)
3	Clements et al. [9] (2010)	27F	CT (TC) → CRT	Local	CT	DOD (12 months)
4	Clements et al. [9] (2010)	54F	Surgery + CRT	Supraclavicular	Surgery + RT	NED (48 months)
5	Oh et al. [8] (2015)	56F	Surgery + CT (PF)	None	N/A	NED (6 months)
6	Abdallah et al. [3] (2016)	64F	CRT (TC)	-	Under treatment	-
7	Isbell et al. [5] (2016)	69F	CRT (VMAT + Cis)	None	N/A	NED (7 months)
8	Isbell et al. [5] (2016)	58F	Surgery + CRT	None	N/A	NED (4 years)
9	Isbell et al. [5] (2016)	47F	None (Refused)	N/A	N/A	DOD (12 months)
10	Matsuzaka et al. [2] (2019)	76F	Surgery + CT (TC)	None	N/A	NED (6 months)
11	Cucinella et al. [4] (2022)	52F	Surgery + CT (TC)	None	N/A	NED (6 months)
12	Matylevich et al. [7] (2024)	62F	Surgery	None	N/A	NED (14 months)
13	Yan and Lin (2024)	59F	Surgery	Local (1 year)	CT (TC)	AWD (PR)
14	Zhang et al. [11] (2025)	49F	Surgery + CT (TC)	None	N/A	NED (12 months)
15	Present case	55F	Surgery	Local (7 months)	Surgery	NED (48 months)

Among the five patients who did not undergo surgical resection, one remained well with no evidence of relapse following chemoradiation with cisplatin. Of the remaining four patients, two received chemoradiation therapy (paclitaxel and carboplatin or cisplatin combined with radiotherapy) and died within one year after the initial diagnosis; the other two patients were undergoing ongoing treatment at the time of analysis. One patient did not undergo any form of therapy and died one year after the initial diagnosis.

In the present case, recurrence was observed seven months after the initial surgery. Based on preoperative imaging, which suggested that the recurrent lesion was isolated and technically resectable, a clinical decision was made to prioritize a second surgical intervention. Complete gross resection was achieved during reoperation. Complete resection likely contributed to a better prognosis and no recurrence in the following four years. Given the lack of sufficient evidence supporting adjuvant therapy, no additional postoperative treatments were administered. These findings suggest that surgical resection is effective in cases of oligo-recurrence in which complete resection is possible.

To the best of our knowledge, this is the first case report describing the successful treatment of retroperitoneal SCC and its local recurrence through surgical resection, achieving long-term survival. A surgical treatment strategy aimed at achieving a “cancer-free” status through reoperation may contribute to improved long-term prognosis.

## Conclusions

We report a case of retroperitoneal SCC of unknown primary origin. Surgical treatment is effective for resectable retroperitoneal SCC, even in cases of local recurrence. Although the findings presented here are based on clinical observations from a single case, our experience demonstrates that complete surgical resection can achieve long-term, disease-free survival, even in the setting of local recurrence. Given the rarity of this disease, further studies are warranted to optimize management strategies and refine patient selection for surgical treatment.

## References

[1] Pack GT, Tabah EJ (1954). Primary retroperitoneal tumors: a study of 120 cases. Int Abstr Surg.

[2] Matsuzaka Y, Yamaguchi K, Moriyoshi K, Takao Y, Takakura K, Konishi I (2019). Primary retroperitoneal squamous cell carcinoma: a case report with review of the literature. Int Cancer Conf J.

[3] Ahdallah R, Morse B, Hakam A, Shahzad MM (2016). Pelvic squamous cell carcinoma of unknown primary: a case report and review of the literature. Eur J Gynaecol Oncol.

[4] Cucinella G, Sozzi G, Di Donna MC, Unti E, Mariani A, Chiantera V (2022). Retroperitoneal squamous cell carcinoma involving the pelvic side wall arising from endometriosis: a case report. Gynecol Obstet Invest.

[5] Isbell A, Fields EC (2016). Three cases of women with HPV-related squamous cell carcinoma of unknown primary in the pelvis and retroperitoneum: a case series. Gynecol Oncol Rep.

[6] Yan H, Lin SD (2024). Case report: HPV related pelvic retroperitoneal squamous cell cancer of unknown primary presenting as ovary neoplasm. Int J Surg Case Rep.

[7] Matylevich OP, Kurchankou MA, Kopsсhaj PA, Schmeler KM (2024). HPV-related metastatic retroperitoneal pelvic squamous cell carcinoma of unknown primary origin in a patient previously treated for endometrial cancer. Int J Surg Case Rep.

[8] Oh HJ, Park EH, Lee YB (2015). HPV-related retroperitoneal squamous cell carcinoma of unknown primary: a case report. Cancer Res Treat.

[9] Clements A, Euscher E, Lacour R, Merritt W, Klopp A, Ramondetta L (2010). The presence of human papillomavirus or p16 in six cases of retroperitoneal carcinoma. Obstet Gynecol.

[10] Khalil AM, Shabb NS, Hourani MH, Shamseddine AI, El-Hajj MI, Seoud AF (2005). Primary squamous cell carcinoma of the pelvic retroperitoneum presenting as an adnexal mass: a case report. J Obstet Gynaecol.

[11] Zhang F, Gui Y, Zhang J (2025). Primary retroperitoneal squamous cell carcinoma: a case report. Oncol Lett.

